# Supplementary material for: Oscillatory ERK Signaling and Morphology Determine Heterogeneity of Breast Cancer Cell Chemotaxis via MEK-ERK and p38-MAPK Signaling Pathways
Source: Bioengineering (Basel). 2023 Feb 18;10(2):269. doi: 10.3390/bioengineering10020269 (PMC9952091; doi:10.3390/bioengineering10020269)
Supplement: Supplementary file 1 [file bioengineering-10-00269-s001.zip › Supplementary figure and table.pdf]

Figure S1

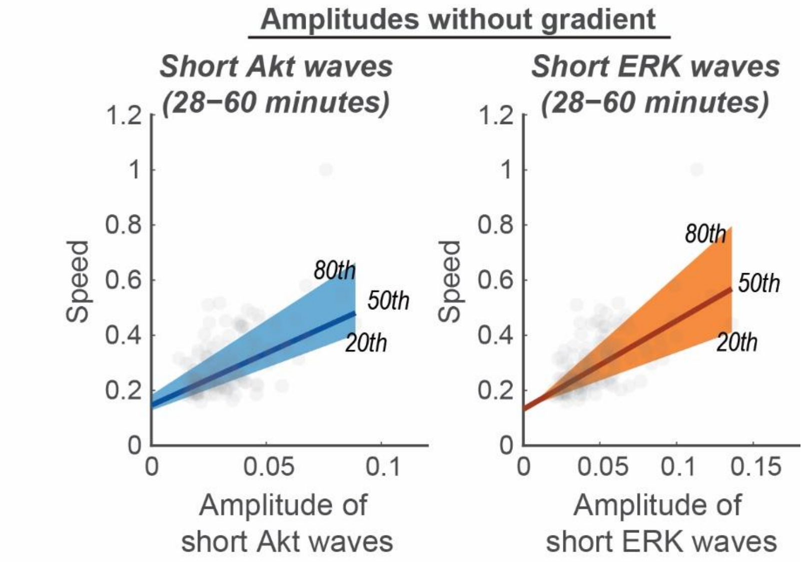

Figure S1: Linear relationship of speed with amplitudes of short signaling waves in conditions without a CXCL12 gradient (n_c = 158). The x-axes of the two plots illustrate the amplitudes of short Akt waves (left) and short ERK waves (right). Their y-axes are all migration speed. The 20th, 50th, and 80th quantile regression results were overlayed to illustrate the relationship between signaling amplitude and speed without CXCL12 gradient.

Figure S2

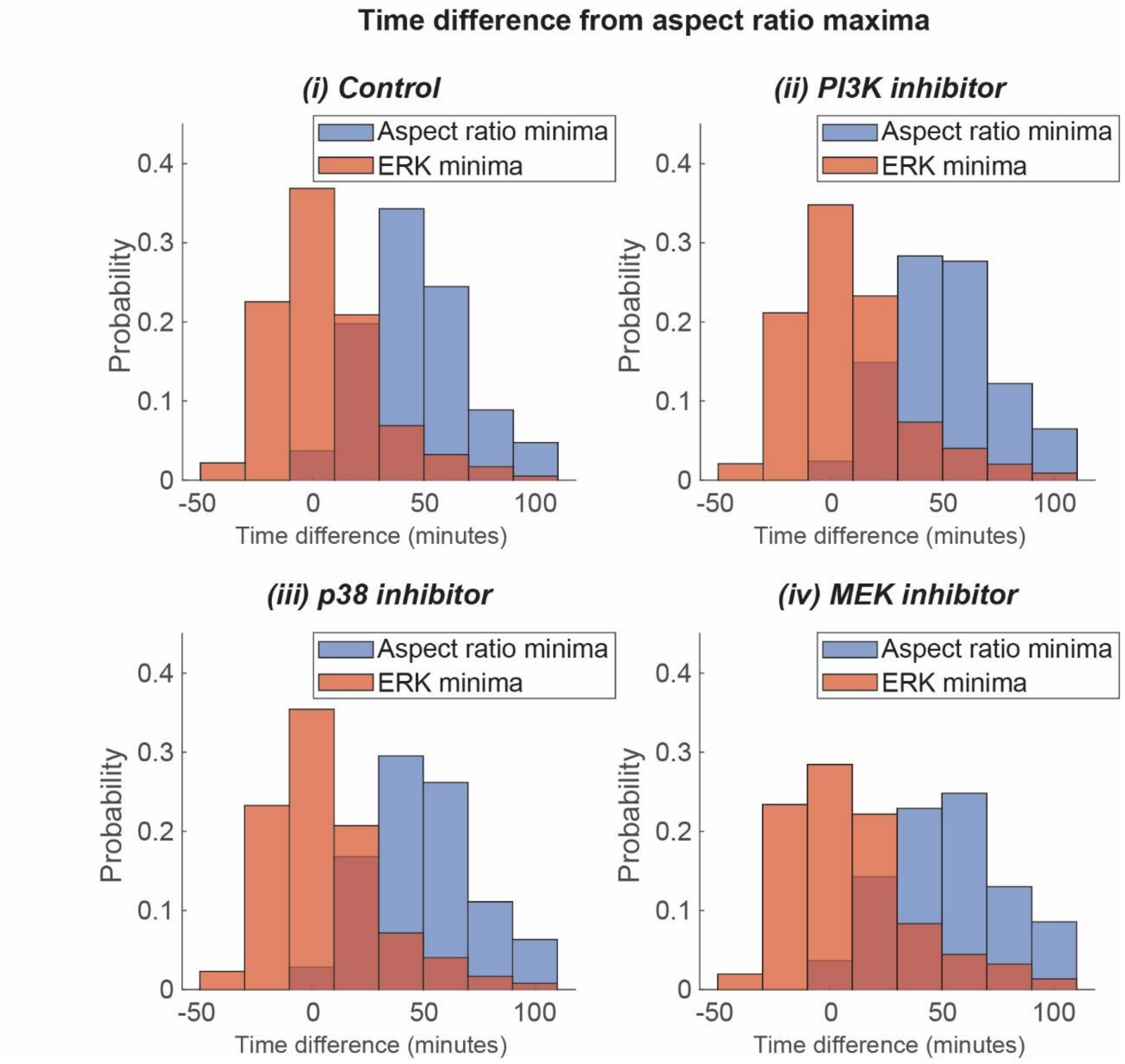

Figure S2: Distributions of aspect ratios and ERK time differences. With the aspect ratio peak aligned, the time differences from this peak to the local minima of aspect ratio and ERK signaling were measured. The overall ERK signal was detrended to eliminate the overall ERK trend and focus on the oscillation patterns. The distributions of the time differences for aspect ratio and ERK minima were illustrated with a histogram in different conditions: (i) Control (no compound), (ii) PI3K inhibitor (100 nM alpelisib), (iii) p38 inhibitor (10 µM SB203580), and (iv) MEK inhibitor (10 nM trametinib).

Table S1

99% confidence intervals for quantile regression of amplitudes vs speed with 100 ng/ml to 0 ng/ml CXCL12 gradient for Figure 1E

	Quantile regression	Equation	Slope	y-intercept
Amplitude of short Akt waves	80th<br>50th<br>20th	y = 8.198x + 0.252<br>y = 6.257x + 0.151<br>y = 3.765x + 0.130	6.224 to 10.173<br>5.182 to 7.332<br>3.235 to 4.295	0.180 to 0.325<br>0.112 to 0.190<br>0.111 to 0.149
Amplitude of short ERK waves	80th<br>50th<br>20th	y = 7.500x + 0.126<br>y = 5.135x + 0.109<br>y = 2.948x + 0.124	6.712 to 8.289<br>4.617 to 5.653<br>2.605 to 3.291	0.083 to 0.170<br>0.080 to 0.137<br>0.105 to 0.142
Amplitude of long Akt waves	80th<br>50th<br>20th	y = 2.207x + 0.450<br>y = 2.214x + 0.275<br>y = 1.768x + 0.182	0.924 to 3.489<br>1.468 to 2.959<br>1.463 to 2.072	0.392 to 0.508<br>0.246 to 0.304<br>0.169 to 0.194
Amplitude of long ERK waves	80th<br>50th<br>20th	y = 2.178x + 0.406<br>y = 2.403x + 0.232<br>y = 1.553x + 0.174	1.368 to 2.987<br>1.945 to 2.861<br>1.286 to 1.821	0.358 to 0.454<br>0.203 to 0.261<br>0.160 to 0.188
Amplitude of aspect ratio	80th<br>50th<br>20th	y = 0.198x + 0.390<br>y = 0.175x + 0.227<br>y = 0.120x + 0.169	0.118 to 0.278<br>0.131 to 0.219<br>0.100 to 0.140	0.320 to 0.461<br>0.193 to 0.261<br>0.153 to 0.186

Table S1: Quantile regression of amplitudes vs speed with 100 ng/mL to 0 ng/mL CXCL12 gradient for Figure 1E. Regression equation, 99% confidence intervals for slope and y-intercept are listed in the table for different amplitudes: short Akt waves, short ERK waves, long Akt waves, long ERK waves, and aspect ratio.

Table S2

99% confidence intervals for quantile regression of amplitudes vs speed **without CXCL12 gradient** for Figure 2E and S1

	Quantile regression	Equation	Slope	y-intercept
Amplitude of short Akt waves	80th<br>50th<br>20th	y = 5.418x + 0.183<br>y = 3.775x + 0.146<br>y = 3.098x + 0.127	2.901 to 7.935<br>2.704 to 4.846<br>2.192 to 4.005	0.083 to 0.283<br>0.108 to 0.184<br>0.096 to 0.159
Amplitude of short ERK waves	80th<br>50th<br>20th	y = 4.963x + 0.120<br>y = 3.214x + 0.131<br>y = 2.030x + 0.135	3.744 to 6.181<br>2.356 to 4.072<br>1.565 to 2.495	0.060 to 0.181<br>0.090 to 0.173<br>0.113 to 0.156
Amplitude of long Akt waves	80th<br>50th<br>20th	y = 0.747x + 0.340<br>y = 1.213x + 0.232<br>y = 0.410x + 0.200	-1.435 to 2.930<br>0.146 to 2.279<br>-0.350 to 1.170	0.256 to 0.425<br>0.198 to 0.267<br>0.173 to 0.228
Amplitude of long ERK waves	80th<br>50th<br>20th	y = 0.568x + 0.331<br>y = 1.201x + 0.223<br>y = 0.526x + 0.191	-1.280 to 2.416<br>0.439 to 1.962<br>-0.008 to 1.060	0.238 to 0.425<br>0.188 to 0.258<br>0.167 to 0.215
Amplitude of aspect ratio	80th<br>50th<br>20th	y = 0.112x + 0.292<br>y = 0.135x + 0.208<br>y = 0.110x + 0.166	-0.028 to 0.252<br>0.069 to 0.200<br>0.069 to 0.151	0.216 to 0.368<br>0.168 to 0.248<br>0.142 to 0.190

Table S2: Quantile regression of amplitudes vs speed without CXCL12 gradient for Figure 2E and S1. Regression equation, 99% confidence intervals for slope and y-intercept are listed in the table for different amplitudes: short Akt waves, short ERK waves, long Akt waves, long ERK waves, and aspect ratio.

Table S3

99% confidence intervals for quantile regression of amplitudes vs speed
with 15 ng/ml to 0 ng/ml EGF gradient for Figure 3D

	Quantile regression	Equation	Slope	y-intercept
Amplitude of short Akt waves	80th<br>50th<br>20th	y = 5.808x + 0.415<br>y = 5.785x + 0.272<br>y = 4.568x + 0.189	4.549 to 7.066<br>4.834 to 6.736<br>3.464 to 5.671	0.356 to 0.474<br>0.228 to 0.317<br>0.136 to 0.242
Amplitude of short ERK waves	80th<br>50th<br>20th	y = 5.193x + 0.325<br>y = 5.004x + 0.197<br>y = 3.372x + 0.174	4.296 to 6.089<br>4.342 to 5.667<br>2.633 to 4.111	0.260 to 0.390<br>0.150 to 0.243<br>0.121 to 0.226

Table S3: Quantile regression of amplitudes vs speed with 15 ng/mL to 0 ng/mL EGF gradient for Figure 3D. Regression equation, 99% confidence intervals for slope and y-intercept are listed in the table for different amplitudes: short Akt waves and short ERK waves.

Table S4

99% confidence intervals for quantile regression of ERK difference, aspect ratio difference, nuclear polarization, and average cell speed for Figure 4C,D,F

	Quantile regression	Equation	Slope	y-intercept
ERK difference vs aspect ratio difference	80th	y = -1.797x + 1.070	-2.107 to -1.488	1.019 to 1.120
	50th	y = -1.389x + 0.431	-1.561 to -1.217	0.404 to 0.458
	20th	y = -1.000x + 0.096	-1.079 to -0.921	0.082 to 0.111
ERK difference vs nuclear polarization	80th	y = -0.223x + 0.307	-0.270 to -0.177	0.299 to 0.315
	50th	y = -0.122x + 0.206	-0.148 to -0.096	0.202 to 0.210
	20th	y = -0.085x + 0.144	-0.106 to -0.063	0.140 to 0.148
Aspect ratio difference vs nuclear polarization	80th	y = 0.137x + 0.223	0.130 to 0.144	0.216 to 0.230
	50th	y = 0.079x + 0.171	0.073 to 0.084	0.166 to 0.177
	20th	y = 0.039x + 0.127	0.035 to 0.044	0.123 to 0.132
Average cell speed vs nuclear polarization	80th	y = 0.192x + 0.231	0.145 to 0.238	0.206 to 0.256
	50th	y = 0.094x + 0.170	0.074 to 0.114	0.160 to 0.180
	20th	y = 0.057x + 0.123	0.039 to 0.074	0.115 to 0.132

Table S4: Quantile regression of ERK difference, aspect ratio difference, nuclear polarization, and average cell speed for figure 4C,D,F. Regression equation, 99% confidence intervals for slope and y-intercept are listed in the table.

Table S5

VSI results for different drug conditions

Sample Name	D (L^2/ T)	vx (L/T)	vy (L/T)
Control1	29.600	-0.193	0.050
Control2	29.700	-0.153	0.095
PI3Ki	32.500	-0.165	0.015
p38i	25.200	-0.077	-0.049
MEKi	23.300	-0.015	0.037

Table S5: Variational system inference results for different drug conditions. D, vx, and vy are the diffusivity and the advective velocities in x and y directions respectively.
