# Supplementary material for: Oscillatory ERK Signaling and Morphology Determine Heterogeneity of Breast Cancer Cell Chemotaxis via MEK-ERK and p38-MAPK Signaling Pathways
Source: Bioengineering (Basel). 2023 Feb 18;10(2):269. doi: 10.3390/bioengineering10020269 (PMC9952091; doi:10.3390/bioengineering10020269)
Supplement: Supplementary file 1 [file bioengineering-10-00269-s001.zip › supplementary information.pdf]

Supplementary information

1 Variational System Inference

The general strong form for the Advection-Diffusion PDE is

$$\frac{\partial C}{\partial t} = D \cdot \Delta C - \nabla \cdot C[v_x, v_y] \tag{1}$$

where D, v_x and v_y are the Diffusivity and the Advective velocities in x and the y directions, respectively.

$$\begin{aligned}\int_\Omega \frac{\partial C}{\partial t} w d\Omega &= \int_\Omega \left(\nabla \cdot (D\nabla C) - \nabla \cdot (C\boldsymbol{v})\right) w d\Omega \\ &= \int_\Omega \left(-D\nabla w \cdot \nabla C + C\boldsymbol{v} \cdot \nabla w\right) w d\Omega + \int_{\partial\Omega} w \left(D\nabla C - C\boldsymbol{v}\right).\boldsymbol{n} ds \end{aligned} \tag{2}$$

where $\boldsymbol{w}$ represents the weighting function (an arbitrary variation on the density field). Following the standard procedure in finite element analysis (FEA) computations of discretizing the domain and accounting for the arbitrariness of the weighting function's degrees of freedom, Equation (2) leads to a set of algebraic equations written as the residual vector, $\boldsymbol{R} = \boldsymbol{0}$ where the residue is given as :

$$\boldsymbol{R} = -\boldsymbol{A}_e \left[\int_{\Omega_e} \left(\frac{\partial C}{\partial t}\boldsymbol{N} + D\nabla \boldsymbol{N} \cdot \nabla C - C\boldsymbol{v} \cdot \nabla \boldsymbol{N}\right) d\Omega - \int_{\partial\Omega_e} \boldsymbol{N} \left(D\nabla C - C\boldsymbol{v}\right).\boldsymbol{n} ds\right] \tag{3}$$

where $\boldsymbol{A}_e$ is the FEM assembly operation and $\boldsymbol{N} = [N^1, \cdots, N^n_{nel}]$ is the vector of interpolation functions in an element. The residue can be succinctly written as:

$$\boldsymbol{R}(D, v_x, v_y) = y - \left[\Xi_1, \Xi_2, \Xi_3\right] \begin{bmatrix} D \\ v_x \\ v_y \end{bmatrix}$$

where

$$y = -\boldsymbol{A}_e \int_{\Omega_e} \frac{\partial C}{\partial t} \boldsymbol{N} d\Omega$$

$$\Xi_1 = \boldsymbol{A}_e \left[\int_{\Omega_e} \nabla \boldsymbol{N} \cdot \nabla C d\Omega - \int_{\partial\Omega_e} \boldsymbol{N} \nabla C . \boldsymbol{n} ds \right]$$

$$\Xi_2 = \boldsymbol{A}_e \left[\int_{\Omega_e} -C \frac{\partial N}{\partial x} d\Omega + \int_{\partial\Omega_e} N C n_x ds \right]$$

$$\Xi_3 = \boldsymbol{A}_e \left[\int_{\Omega_e} -C \frac{\partial N}{\partial y} d\Omega + \int_{\partial\Omega_e} N C n_y ds \right]$$

The Variational System Identification problem is then posed as the following minimization problem:

$$D, v_x, v_y = \underset{\widetilde{D}, \widetilde{v}_x, \widetilde{v}_y}{\arg\min} |\boldsymbol{R}(\widetilde{D}, \widetilde{v}_x, \widetilde{v}_y)|^2 \quad (4)$$
